# 

**Published:** 1904-01-09

**Authors:** 


					?"The standard of highest purity."?Lancet. January 9th, 1904.
?JBURY'S U CADBURY'S
COCOA m, MM . COCOA , ? ?, -
"A PERFECT FOOD."  NO DRUGS OR ALKAL;r^\$kwftV
Western Infirmary
No. 902. Vol. XXXY. [fgi!] Saturday, Jan. 9th, 1904. [Suhscnptpion Terms,] pR|(;E THREEpENCEa

				

